# A highly processive actinobacterial topoisomerase I – thoughts on *Streptomyces*’ demand for an enzyme with a unique C-terminal domain

**DOI:** 10.1099/mic.0.000841

**Published:** 2019-08-07

**Authors:** Marcin J. Szafran, Agnieszka Strzałka, Dagmara Jakimowicz

**Affiliations:** ^1^​ Laboratory of Molecular Microbiology, Faculty of Biotechnology, University of Wroclaw, Wroclaw, Poland

**Keywords:** *Streptomyces*, topoisomerase I, chromosome topology

## Abstract

Topoisomerase I (TopA) is an essential enzyme that is required to remove excess negative supercoils from chromosomal DNA. Actinobacteria encode unusual TopA homologues with a unique C-terminal domain that contains lysine repeats and confers high enzyme processivity. Interestingly, the longest stretch of lysine repeats was identified in TopA from *Streptomyces *, environmental bacteria that undergo complex differentiation and produce a plethora of secondary metabolites. In this review, we aim to discuss potential advantages of the lysine repeats in *Streptomyces* TopA. We speculate that the chromosome organization, transcriptional regulation and lifestyle of these species demand a highly processive but also fine-tuneable relaxase. We hypothesize that the unique TopA provides flexible control of chromosomal topology and globally regulates gene expression.

## Introduction

Actinobacteria are the largest (130 genera) and most phylogenetically distinct group of bacteria, exhibiting remarkably diverse environmental niches, life cycles and cell morphologies, varying from unicellular rods to multicellular hyphae [1]. The actinobacteria that attract the most research interest include pathogenic *Mycobacteria* and antibiotic-producing *Streptomyces* [2]. *Streptomyces* not only produce a plethora of pharmacologically valuable secondary metabolites, but also, because of their complex morphological differentiation, they are interesting model organisms in studies on bacterial development and gene regulation [3–7]. Complex regulatory cascades govern crucial switches during *Streptomyces* sporulation, e.g. the emergence of aerial hyphae or the cessation of their growth followed by the generation of spore chains [8–10]. Interestingly, the differentiation of *Streptomyces* is accompanied by the activation of secondary metabolite gene clusters that are also controlled by multi-layered regulatory pathways [8–11]. While it is recognized that chromosome topology acts as a global transcriptional regulator in various eukaryotes and prokaryotes [12, 13], the impact of chromosome spatial structure on gene regulation and secondary metabolite production in *Streptomyces* has just been established [5, 14–18].

Interestingly, *Streptomyces* undergo profound changes in their chromosome organization during their complex life cycle [19, 20]. Moreover, they are distinct among bacteria due to the presence of multiple copies of linear chromosomes in their elongated hyphal cells. While throughout vegetative growth, chromosomes remain uncondensed and visibly unseparated in hyphal cells, they become highly compacted during the formation of unigenomic spores. As in other bacteria, *Streptomyces *’ chromosome topology is controlled by several proteins, including nucleoid-associated proteins (NAPs) [15, 21, 22], condensins [23, 24] and topoisomerases [14]. Interestingly, topoisomerase I (TopA), *Streptomyces *’ major DNA relaxase, exhibits unusually high processivity [25, 26]. It is intriguing that *Streptomyces* require an extremely processive DNA relaxase and in this review we discuss the properties of this unique enzyme in relation to *Streptomyces *’ environment, growth features and chromosome topology.

### Bacterial topoisomerases

The discovery of topoisomerases in the early 1970s answered a question that had been open for almost 2 decades – how do cells deal with chromosomal topological problems that occur during the unwinding of the DNA double helix and are manifested by an accumulation of DNA supercoils [27, 28]. While appropriate DNA supercoiling compacts chromosomes and contributes to packaging of genetic material in the limited intracellular space, it also facilitates the unwinding of the DNA double helix that is required for the initiation of transcription and replication [29, 30]. On the other hand, an excess of DNA supercoils inhibits the progress of replication and transcription and therefore is detrimental to cell growth [31, 32]. Thus, the appropriate level of negative DNA supercoiling, also named topological homeostasis or supercoiling balance, needs to be preserved to allow for the progression of the DNA transactions, while maintaining chromosome compaction.

The supercoiling balance is controlled by topoisomerases, the enzymes that transiently break and re-join DNA strands to remove and add supercoils to the DNA double helix. Based on structural differences and the mechanism of action, the topoisomerases are classified into two types [33, 34]. Type I topoisomerases primarily function as monomers (with the exception of heterodimeric reverse gyrase [35]) that cut a single DNA strand and re-ligate it in an ATP-independent manner. By contrast, type II topoisomerases, which function as dimers or heterotetramers, cut both DNA strands and hydrolyze ATP to induce conformational changes that allow the transfer of the intact DNA duplex throughout the cleaved DNA double helix [35–37]. In general, the predominant function of bacterial type I topoisomerases is to remove negative supercoils (although some type I enzymes are also able to remove positive supercoils), while bacterial type II enzymes are responsible for the removal of the positive supercoils [34, 38]. Thus, due to their opposing activities, both types of enzymes are required for the survival of every bacterial cell. Although the minimal set of topoisomerases in bacteria is limited to just two topoisomerases, TopA and gyrase, most species possess more than one topoisomerase of each type, such as an additional type I enzyme [topoisomerase III (TopB)] and/or type II enzyme [topoisomerase IV (ParCE)]. These additional enzymes are involved in distinct DNA transaction processes (DNA repair and recombination, sister chromosome decatenation, DNA relaxation and compaction), but are still able to partially complement the cellular functions of the main topoisomerases [38–41].

**Fig. 1.**
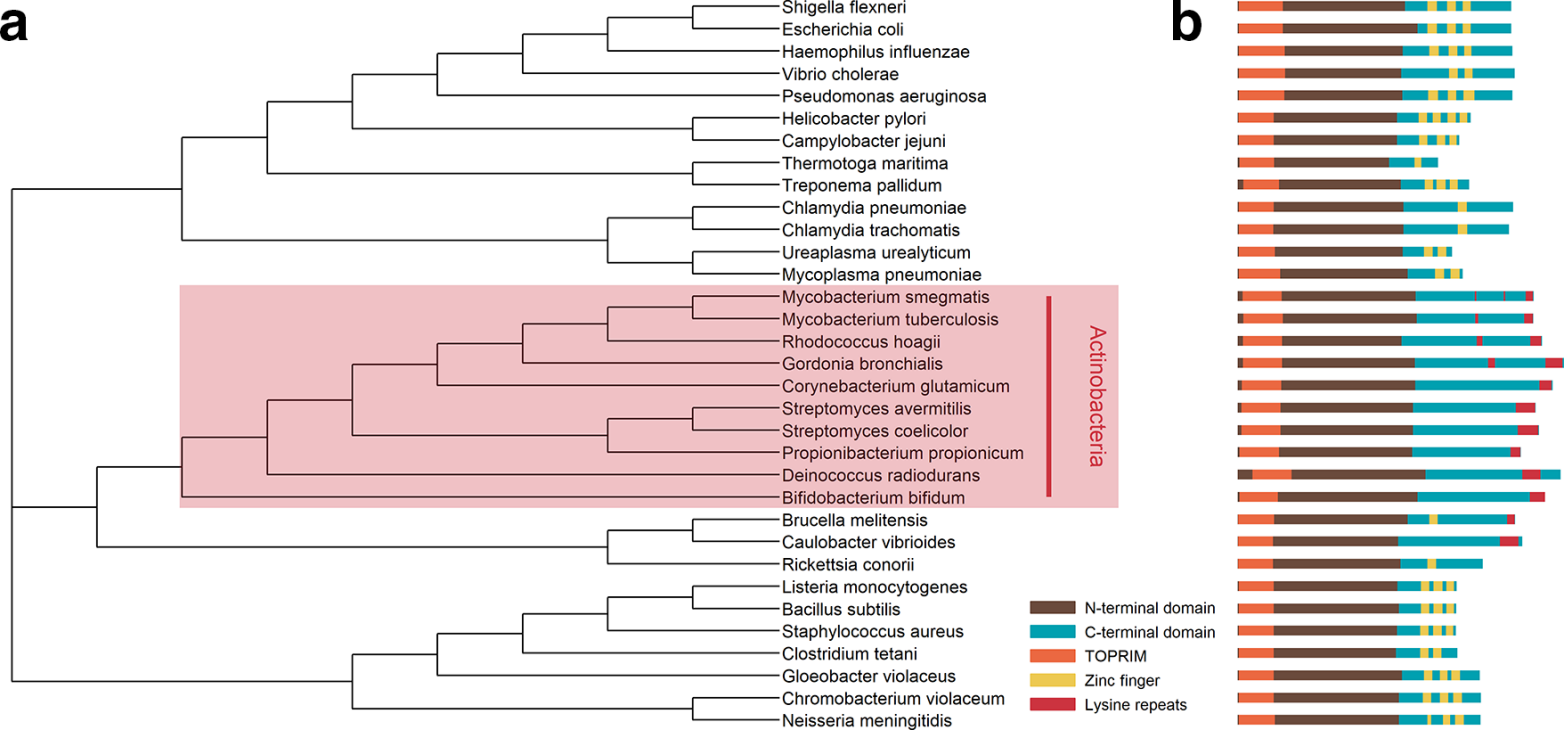
Comparison of the primary structures of TopA homologues. (a) A phylogenetic tree (constructed using ClustalW in the R msa package [97]) of TopA homologues in selected bacteria species. (b) The primary structures of bacterial TopA with N-terminal domain, C-terminal domain, TOPRIM motif, zinc fingers and lysine repeats indicated.

As in other bacteria, actinobacterial assortment of topoisomerases varies between particular groups and even differs between closely related species. Although some mycobacteria encode only the minimal set of enzymes, encompassing TopA and gyrase (e.g. *Mycobacterium tuberculosis* and *Mycobacterium leprae *), other species genomes (﻿e.g. *Mycobacterium smegmatis* and *Mycobacterium avium *) contain genes encoding the additional topoisomerases. They include a poxvirus-like type I topoisomerase, which was presumably acquired as a result of horizontal gene transfer, and a type II topoisomerase distinct from topoisomerase IV, which is not essential but supports the decatenation of newly replicated chromosomes [42–44]. Interestingly, *Streptomyces* also encode topoisomerase IV, but, due to the linearity of their chromosomes, its activity is not required for chromosome separation [45]. The majority of *Streptomyces* species possess only one topoisomerase of type I; however, a gene encoding additional poxvirus-like type I topoisomerase is present in the genomes of some species (e.g. *Streptomyces venezuelae *). Although exhibiting a highly diverged assortment of topoisomerases, all actinobacteria contain characteristic TopA proteins (Fig. 1).

### Unique features of actinobacterial TopA

Actinobacterial TopAs possess two distinct features that distinguish them from other bacterial topoisomerase I homologues: a unique C-terminal domain and high supercoil removal processivity [25, 46]. Additionally, these enzymes exhibit other unusual species-specific properties. For instance, unlike most type I topoisomerases, *M. smegmatis* TopA was shown to have a DNA sequence preference, exhibiting strong topoisomerase site (STS) recognition [47]. On the other hand, Bao and Cohen identified *Streptomyces coelicolor* TopA as a part of a large nucleoprotein complex associated with the ends of linear chromosomes. Moreover, the same studies showed that TopA exhibited *in vitro* reverse transcriptase activity that was dependent on two conserved aspartic acid residues located within the N-terminal domain [48]. While the classical topoisomerase activity studies on *M. smegmatis* TopA demonstrated its high processivity, single-molecule analysis also reconfirmed this observation for *S. coelicolor* TopA. Application of a magnetic trap and a DNA fragment that was up to 51 kb long made it possible to measure the number of supercoils removed in a single reaction burst (up to 150 compared to approximately 20 for *Escherichia coli* enzymes), as well as the supercoil removal velocity (the number of supercoils removed per second, which for *S. coelicolor* TopA and *E. coli* TopA were within the same range, 8.0 and 3.3 Lk s^−1^, respectively) [25, 49, 50]. Thus, the processivity of *S. coelicolor* TopA exceeds that of any other studied type I topoisomerase. Studies on truncated *M. smegmatis* and *S. coelicolor* TopA homologues revealed that their high processivity is conferred by their unique C-terminal domains [25, 26, 46].

Similarly to all other TopA homologues, actinobacterial enzymes consist of two domains: the N-terminal domain (NTD), which contains the catalytical tyrosine residue and topoisomerase/primase (TOPRIM) motif, and the shorter C-terminal domain (CTD) [25, 46]. Importantly, unlike other TopA homologues (but similarly to TopB enzymes), the actinobacterial TopA CTD lacks zinc finger motifs, which in *E. coli* TopA were shown to be responsible for binding single-stranded DNA [51]. The distinctive feature of the actinobacterial TopA CTD is the presence of multiple degenerate repeats enriched in lysine residues [lysine repeats (LRs)], which resemble sequences that are present in eukaryotic histone H1 [25, 26, 52] (Fig. 1). Interestingly, sequence analyses identified LRs that were also in TopA homologues from *Caulobacter crescentus* and *Bordetella pertussis *, which, similarly to actinobacteria, possess GC-rich genomes (GC content 67 %) [26]. Nevertheless, *Streptomyces* TopA homologues contain the longest (approximately 12 repeats within the 70 amino acid fragment) stretch of LRs among TopAs, which is followed by two conserved acidic amino acids [26]. In *M. smegmatis *, apart from the LRs at the C-terminus, two additional, shorter fragments rich in basic amino acids were also identified in TopA CTD [46]. Although partial crystal structure is available for *M. tuberculosis* TopA, it only delivered information on a fragment of CTD that did not include LRs, thus the structure of *Streptomyces *’ LR-rich fragment is based solely on prediction, which suggests the formation of an alpha helix [25, 53]. Interestingly, our search for LR motifs in *Streptomyces *’ proteome indicated that similar LRs are present in several DNA-binding proteins, i.e. sigma factor HrdB, DNA repair Ku protein or nucleoid-associated protein HupS (* E. coli* HU homologue) [21, 26]. In fact, the lysine-rich C-terminal domains of Ku protein and mycobacterial HupS homologue (HupB) were shown to be required for its interaction with DNA [54, 55].

Although C-terminally truncated *M. smegmatis* TopA (NTD) was shown to bind, cut and religate DNA strands, it was not capable of promoting DNA relaxation. Like *M. smegmatis *’s truncated enzyme, *S. coelicolor* TopA NTD was demonstrated to be insufficient for supercoil removal [25, 56]. Notably, for both enzymes, *M. smegmatis* and *S. coelicolor* TopA, it was shown that CTDs and NTDs could be separated and mixed to restore enzyme activity, suggesting a direct interaction between these domains [26, 56]. *M. smegmatis* TopA with truncations of CTD exhibited diminished DNA binding and decreased DNA relaxation activity due to the impaired strand passage, which is a critical step of catalyzed reaction. Thus CTD was demonstrated to provide an additional DNA-binding domain, performing a similar function to zinc finger motifs in *E. coli* [46]. Studies of *S. coelicolor* TopA–DNA binding showed that although the enzyme lacking LRs exhibited high DNA-binding affinity, it was more likely to disassociate from DNA [26]. Moreover, single-molecule analysis revealed that the processivity (the number of supercoils removed in one enzymatic burst) of the LR-truncated TopA decreased dramatically. However, in the case of *S. coelicolor* TopA, the velocity of reaction, calculated as the number of the supercoils removed per second, was unchanged by the LR truncation, suggesting that in *Streptomyces* TopA LRs are not involved in the reaction itself [26]. Consequently, it was suggested that the LRs in *Streptomyces* TopAs stabilize enzyme–DNA complexes during reactions [26]. The stability of the enzyme–DNA complex was hypothesized to result from the interaction between the NTD and CTD with LRs and terminal acidic amino acids potentially involved in the binding of NTD. Such an interaction is supposed to lead to the formation of a clamp around the DNA that ensures high TopA processivity [26].

What were the evolutionary pressures that selected for the unique properties in actinobacterial TopA and promoted the increased number of LRs in *Streptomyces* topoisomerase, conferring unusual enzyme processivity? Although the actinobacteria are a remarkably broad and varied group of bacteria, they all have GC-rich genomes. While LRs are a common feature of actinobacterial TopAs, their presence in the topoisomerases of other GC-rich bacteria and in other DNA-binding proteins [26] suggests their particular significance for the stabilization of protein complexes on GC-rich DNA. This hypothesis raises the question of why GC-rich bacteria require increased stability of the TopA–DNA complex. For type I topoisomerases, the explanation may be their preference for ssDNA as a binding site [26, 57]. Since DNA unwinding in GC-rich genomes is limited and the binding sites for TopA are scarce, the increased TopA–DNA complex stability would be highly advantageous. However, a question that remains unanswered is why there are an increased number of LR motifs in *Streptomyces* TopA compared to other actinobacterial TopA homologues.

### Topological homeostasis in *Streptomyces*

The stabilization of the TopA–DNA complex may not be the only function of the enzyme unique CTD. We hypothesize that the LRs positioned within CTD that increase the stability of the enzyme complex on DNA and its processivity are likely to be involved in regulation of the enzyme activity. Speculatively, the high number of LRs that could be modified to diminish the DNA binding might possibly allow the fine-tuning of *Streptomyces* TopA processivity and enable the rapid control of chromosome supercoiling. This notion is supported by the fact that, while, in most bacteria, the major mechanism for chromosome supercoiling maintenance is based on the transcriptional regulation of the *topA* and *gyrAB* genes, in *Streptomyces* the transcriptional regulation of topoisomerase genes seems to be limited [58–60]. The sensitivity of *Streptomyces* topoisomerase genes to topological changes differs from the transcriptional regulation observed in other bacteria, suggesting the presence of additional regulatory mechanisms.

**Fig. 2.**
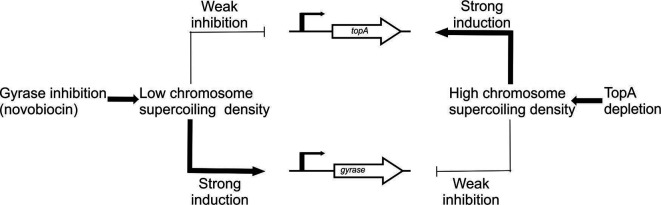
Scheme of the regulation of chromosome supercoiling in *S. coelicolor* by modifications to topoisomerase gene transcription [58].

The susceptibility of gyrase-encoding genes to supercoiling imbalance is highly conserved among bacteria, even though the *gyrA* and *gyrB* genes may be arranged as a single dicistronic operon (* M. smegmatis* [61], *S. coelicolor* [62] and *Borrelia burgdorferi* [63]) or as the two separately transcribed genes (* E. coli* [64] and *Bacillus subtilis* [65]). Similar to mycobacteria, the *Streptomyces gyrA* and *gyrB* genes are arranged in a tandem, with *gyrB* positioned upstream of *gyrA*, suggesting their potential dicistronic transcription, although the organization of their promoter region is unknown. In *S. coelicolor *, *gyrBA* transcription is stimulated by chromosome relaxation, which may result from the inhibition of gyrase with novobiocin (Fig. 2). Surprisingly, the *S. coelicolor gyrBA* operon is insensitive to the increased DNA supercoiling, which in other bacteria typically leads to the reduction of gyrase transcription [58, 59].

In contrast to relaxation-induced gyrase gene regulation, *topA* transcription is induced by increased supercoiling (Fig. 2). Importantly, chromosome relaxation resulting from gyrase inhibition only slightly affects *topA* transcription. This situation is different from the transcriptional regulation observed in the other bacteria, where *topA* transcription is decreased under such conditions [59, 66]. In *E. coli *, *topA* gene transcription is controlled by four promoters, the activities of which change at different growth stages as well as in response to stress conditions, e.g. heat shock. Moreover, at least three *topA* promoters in *E. coli* are sensitive to changes in the overall negative chromosome supercoiling [66]. In contrast to *E. coli *, the number of promoters controlling *topA* transcription in actinobacteria is limited to only two. Whereas both *M. smegmatis topA* promoters are sensitive to supercoiling changes in *S. coelicolor *, only one of the two *topA* promoters (named *topA*p1) is sensitive to alterations in negative DNA supercoiling [58, 67]. A comparative analysis of the *topA*p1 promoter revealed that its −10 and −35 nucleotide sequences resemble those recognized by the housekeeping sigma factor HrdB; however, the spacer region was much shorter (13 bp) than the typical 17–18 bp for *hrdB*-dependent promoters [68]. Interestingly, such a decrease in the sequence length of the spacer has been shown to be a common feature of supercoiling-sensitive promoters [66, 67, 69]. Thus, in actinobacteria, and particularly in *Streptomyces *, although they are more likely exposed to environmental factors that affect chromosome supercoiling, the transcriptional regulation of TopA level appears to be surprisingly less complex than in *E. coli *. Moreover, the *topA* gene is constitutively transcribed during the entire *S. coelicolor* life cycle, suggesting the existence of different mechanisms of TopA activity regulation.

**Fig. 3.**
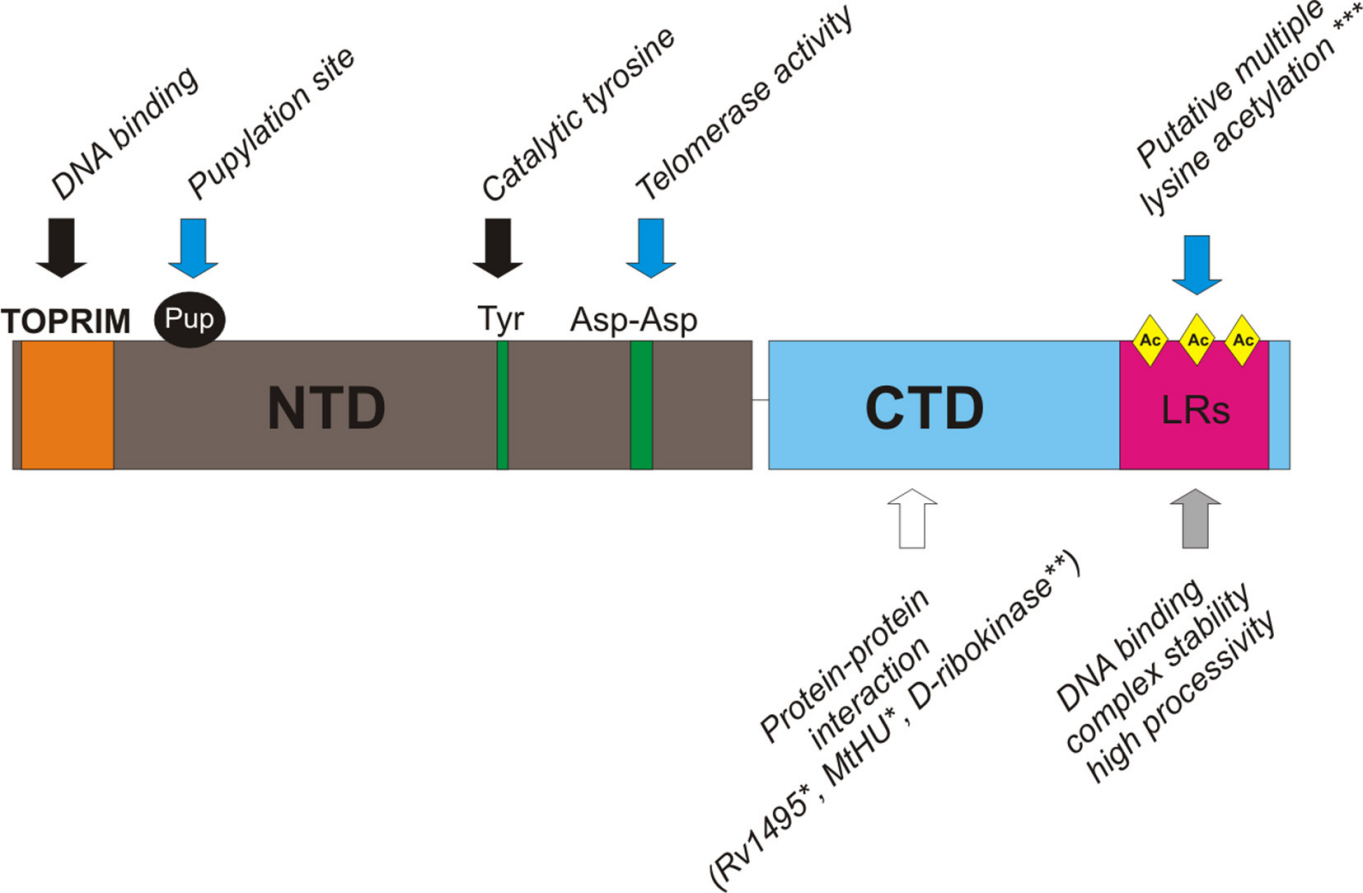
Scheme of *S. coelicolor* TopA domains with important catalytic and putative regulatory residues and regions indicated. The black arrows indicate the conserved topoisomerase I features, the grey arrows indicate characteristic actinobacterial features and the blue arrows indicate *S. coelicolor* TopA-specific features, *** indicates possibly conserved in *Mycobacteria* and the white arrows refer to TopA CTD protein–protein interactions identified in *M. tuberculosis* (*) or *M. smegmatis* (**); see the detailed description in the text.

The circumstantial evidence suggests that the activity of TopA homologues may be regulated posttranslationally by direct interaction with other proteins or by reversible posttranslational modifications (PTMs) (Fig. 3). In various bacteria TopA activity can also be modulated by direct protein–protein interactions or by changes in DNA structure induced by DNA-binding proteins. For example, TopA activity was demonstrated to be affected by RecA in *E. coli *, while in *M. smegmatis* and *S. coelicolor* it is affected by the nucleoid-associated proteins HupB and sIHF, respectively, as well as by a component of a toxin–antitoxin system, MazF, in *M. smegmatis* [15, 70–74]. On the other hand, both *E. coli* and *M. smegmatis* TopA are recruited by RNA polymerase during transcription via a direct interaction mediated by their C-terminal domains, thus promoting TopA activity at specific chromosomal loci [75–77].

Recent proteomic studies suggest that *Streptomyces* TopA may also be a target for a posttranslational modification called pupylation, which is the covalent attachment of prokaryotic ubiquitin-like protein (Pup) [78]. Pupylation is a PTM that is limited to actinobacteria and targets modified proteins for subsequent proteasome degradation [79]. Moreover, the activity of *E. coli* TopA was shown to be affected by reversible lysine acetylation [72]. Importantly, this PTM was also identified in mycobacterial Ku and HupB proteins, in which stretches of lysine residues similar to the LRs present in TopA are targets for modification [80, 81]. The LR acetylation in the *M. tuberculosis* HupB CTD affects the HupB DNA binding [81, 82]. Moreover, modulation of HupB binding to DNA by acetylation/deacetylation was suggested to remodel the mycobacterial chromosome in response to changes in environmental conditions or antibiotic treatments. Our preliminary studies suggest that the LRs in *Streptomyces* TopA are also the likely targets for lysine acetylation (M. Szafran, unpublished). Thus, we speculate that since the high processivity of actinobacterial TopA is dependent on LRs in the C-terminal domain, the protein–protein interactions or PTMs that occur within CTD may constitute a regulatory mechanism that could fine tune enzyme processivity in response to physiological demands or environmental conditions. Since *Streptomyces* are exposed to a variety of stress factors that may affect chromosome topology and require a rapid response, and because the transcriptional regulation of their topoisomerase genes is limited, the other mechanisms are likely to modulate TopA activity. The idea that TopA activity is subject to regulation by posttranscriptional or interaction with other proteins is reinforced by its presence at a constant level during *Streptomyces *’ complex life cycle.

### Requirement for TopA during *Streptomyces *’ life cycle

During *Streptomyces* sporulation their chromosomes undergo profound changes of topology, from being visibly uncondensed in hyphal cells to highly compacted in spores [9, 19, 20]. Chromosome compaction was shown to be assisted by condensin (SMC) and nucleoid-associated proteins, namely, the sporulation-specific HU homologues HupS, sIHF and DpsA [15, 21, 23, 83]. Analysis of *S. coelicolor* and *S. venezuelae* TopA-depleted strains showed that sporulation also requires TopA activity. Severe depletion of TopA not only slowed *Streptomyces* growth, but also led to a ‘white’ phenotype (indicating the absence of pigmented spores) and inhibition of sporulation cell division [14, 84]. Interestingly, lowering TopA processivity did not disturb growth rate but rather delayed the formation of spores and affected the length of spore chains [26]. This result shows that although the level of TopA appears to be constant throughout the life cycle [58], sporulation requires increased TopA processivity.

**Fig. 4.**
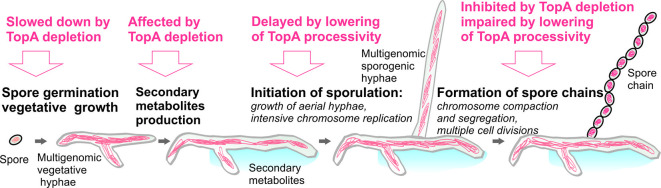
Stages of the *S. coelicolor* life cycle affected by modifications in the level and processivity of TopA

The rapid extension of aerial hyphae at the onset of sporulation is accompanied by intensive chromosome replication. Tens of chromosome are required in the elongated hyphal cell to produce a chain of unigenomic spores, generated by synchronized multiple divisions [8, 85, 86] (Fig. 4). The shortened spore chains produced by an *S. coelicolor* strain with an LR-truncated TopA suggest that intensive chromosome replication requires particularly high TopA processivity. Before aerial hyphae septation, chromosomes are evenly distributed along the hyphal cell by the segregation proteins ParA and ParB [19, 87, 88]. As in other bacterial species that use ParABS system for chromosome segregation, the ParB protein in *Streptomyces* forms segregation complexes (segrosomes) by interacting with numerous DNA sequences called *parS* sites, which are located in proximity to the chromosomal origin of replication (*oriC*) [88]. Remarkably, the distribution and separation of the ParB complexes in *Streptomyces* sporogenic hyphae were found to be impaired by TopA depletion [14, 89]. Taking into account the fact that the *S. coelicolor* chromosome contains an unusually high number of *parS* sites and ParB binding was shown to lead to bridging of distant binding sites, we hypothesize that segrosome formation generates topological tension. Consequently, to enable segrosome separation, this topological tension must be released by TopA [14, 84]. The recruitment of TopA to ParB complexes may be the mechanism by which TopA activity is stimulated, although a direct interaction between TopA and ParB has not been detected. The proposed explanation for the observed inhibition of sporogenic cell division in a TopA-depleted strain may be at least partially due to unsegregated chromosomes. This would indicate the existence of a nucleoid occlusion-like mechanism, which has been previously reported for several bacterial species (e.g. *B. subtilis* and *E. coli *) [84, 87, 90–92]. However, another possible explanation for the impact of TopA depletion on cell division may be changes in the transcription of supercoiling-sensitive genes (SSGs).

In *S. coelicolor *, as in other bacterial species (* E. coli *, *Streptococcus pneumoniae* and *Haemophilus influenzae *), chromosome supercoiling has been shown to function as a global transcriptional regulator [16, 93–95]. The changes in chromosome supercoiling induced by either gyrase or TopA inhibition affects a substantial fraction of genes (7–37 %), which varies among species and assay conditions. Nevertheless, sets of SSGs consistently include those encoding topoisomerases and other proteins involved in DNA transactions [96]. In *Streptomyces *, changes in chromosome supercoiling in a TopA-depleted strain have a profound effect on global gene expression, including sporulation specific regulators such as *whiG* (but not *ftsZ*, as reported for mycobacteria) and genes encoding DNA repair proteins [16]. Interestingly, in *S. coelicolor *, the impact of DNA supercoiling on global gene transcription also encompasses genes involved in secondary metabolite production. TopA depletion was observed to affect the transcription of a large number of genes encoding regulatory protein, which may explain the high overproduction of actinorhodin observed in a TopA-depleted strain [14, 16]. On the other hand, the rapid chromosome relaxation in *S. coelicolor* results in the induction of several secondary metabolite gene clusters, including those involved in the synthesis of coelibactin, as well as the induction of the actII-4-encoding actinorhodin cluster activation protein [16]. These observations suggest that manipulation of DNA supercoiling may potentially be used to induce secondary metabolite production in *Streptomyces *, although the mechanisms by which changes in DNA topology affect secondary metabolism have not yet been fully explored.

### Concluding remarks

Actinobacteria have primarily been studied with respect to *M. tuberculosis* pathogenicity and secondary metabolite production by *Streptomyces *. Recently, mycobacterial unique topoisomerase I has attracted attention due to its potential use as a target for novel anti-tuberculosis antibiotics, while its *Streptomyces* homologue was shown to be required for the progression of the cell cycle and to be involved in global gene regulation [14, 16, 44].

The high enzyme processivity of actinobacterial TopA homologues is believed to be conferred by the LR-enriched C-terminal domain via stabilization of the enzyme–DNA complex. This increased complex stability appears to be advantageous for GC-rich organisms. The elongated C-terminal domains containing LRs are a hallmark of actinobacterial TopAs, although the *Streptomyces* TopA CTD contains more LRs than its homologues in other *Actinobacteria *. One possible reason why *Streptomyces* TopA may require an increased number of LRs and enhanced processivity is that it has a complex life cycle that demands the processing of multiple copies of chromosomes, especially during sporulation. The formation of segregation complexes by ParB was suggested to generate the topological tension that is presumably relieved by the recruitment of TopA. However, the constitutive level of TopA expression observed during the *S. coelicolor* differentiation reinforces the potential involvement of posttranslational regulation via PTMs or direct protein–protein interactions. Such regulation could be beneficial during changes in chromosome topology and during the complex life cycle, as well as in response to environmental stress.

The rapid changes in chromosome topology impact on global gene transcription, potentially indicating that targeted changes in chromosome supercoiling may be used to optimize secondary metabolite production. However, the industrial application of supercoiling-modified strains requires a better understanding of the complex mechanisms that restore and maintain the optimal level of chromosome supercoiling.
